# From Discrete to Continuous Modeling of Lymphocyte Development and Plasticity in Chronic Diseases

**DOI:** 10.3389/fimmu.2019.01927

**Published:** 2019-08-20

**Authors:** Jennifer Enciso, Rosana Pelayo, Carlos Villarreal

**Affiliations:** ^1^Centro de Investigación Biomédica de Oriente, Instituto Mexicano del Seguro Social, Mexico City, Mexico; ^2^Programa de Doctorado en Ciencias Biomédicas, Universidad Nacional Autónoma de México, Mexico City, Mexico; ^3^Centro de Ciencias de la Complejidad, Universidad Nacional Autónoma de México, Mexico City, Mexico; ^4^Departamento de Física Cuántica y Fotónica, Instituto de Física, Universidad Nacional Autónoma de México, Mexico City, Mexico

**Keywords:** lymphocytes, chronic diseases, boolean, fuzzy logic, computational modeling

## Abstract

The molecular events leading to differentiation, development, and plasticity of lymphoid cells have been subject of intense research due to their key roles in multiple pathologies, such as lymphoproliferative disorders, tumor growth maintenance and chronic diseases. The emergent roles of lymphoid cells and the use of high-throughput technologies have led to an extensive accumulation of experimental data allowing the reconstruction of gene regulatory networks (GRN) by integrating biochemical signals provided by the microenvironment with transcriptional modules of lineage-specific genes. Computational modeling of GRN has been useful for the identification of molecular switches involved in lymphoid specification, prediction of microenvironment-dependent cell plasticity, and analyses of signaling events occurring downstream the activation of antigen recognition receptors. Among most common modeling strategies to analyze the dynamical behavior of GRN, discrete dynamic models are widely used for their capacity to capture molecular interactions when a limited knowledge of kinetic parameters is present. However, they are less powerful when modeling complex systems sensitive to biochemical gradients. To compensate it, discrete models may be transformed into regulatory networks that includes state variables and parameters varying within a continuous range. This approach is based on a system of differential equations dynamics with regulatory interactions described by fuzzy logic propositions. Here, we discuss the applicability of this method on modeling of development and plasticity processes of adaptive lymphocytes, and its potential implications in the study of pathological landscapes associated to chronic diseases.

## 1. Introduction

The extensive accumulation of data from short and large-scale experiments involving a wide spectrum of biological functions of B and T lymphocytes in both, normal and pathological scenarios, has inspired an intensive research on molecular events leading to their early development, plasticity and emergency differentiation. As a result, the construction of regulatory networks has become a resourceful tool for the systems-level analyses of cell fate decisions through interconnection of molecular elements, such as biochemical signals provided by the microenvironment (e.g., cytokines, growth factors, transmembrane ligands, antigens, etc.) and transcriptional modules underlying the regulation of lineage-specific gene expression. Getting insights into the dynamical behavior of regulatory networks in biology requires simulation as continuous or discrete models (1). Discrete modeling, represented by Boolean and multi-valued network models, has been useful in differentiation processes of adaptive B and T lymphocytes (2–8), for molecular switching in cellular specification (9), for the prediction of microenvironment-dependent cell plasticity (6, 10), and for the analyses of signaling events occurring downstream activation of antigen recognition receptors (11, 12). Moreover, Boolean algebra has been used in cytometry to create combined gates for the identification and selection of cellular subsets and lymphoid phenotyping (13). Nevertheless, the utility of discrete models is limited as they cannot predict outcomes from quantitative biological experiments when working on phenomena sensitive to graded expression of transcription factors or biochemical gradients. This is the case of most diseases where lymphocytes are involved and non-discrete fluctuations in the microenvironment may influence cell differentiation and plasticity, affecting immune responses at the progression of chronic pathologies, such as lymphoproliferative disorders, tumor growth, diabetes, cardiovascular, and chronic respiratory diseases, among others. Discrete models might be then transformed into differential equations to allow a dynamical analyses of regulatory networks, as transformed continuous models, with potential implications in lymphoid cell- associated pathologies (14–17).

Here we propose the fuzzy logic transformation of a discrete model into a continuous model to compensate their disadvantages and to simulate biological systems with a well-known network architecture strongly influenced by concentration-dependent cues (Table 1).

**Table 1:** Mathematical dynamic modeling subtypes: advantages and disadvantages.

**Dynamic modeling**	**Advantages**	**Disadvantages**	**Type of application**
Discrete	•Simulation of large-scale biological systems (e.g., hundreds of components). •Simulation of biological systems with scarce knowledge of kinetic parameters and mechanistic details. •Useful for qualitative dynamic descriptions of system behaviors. •Large quantities of qualitative information available in published literature and high-throughput experiments.	•Assumption of discretization for all components of the system. •Attractors are hardly comparable to experimental information that contains graded expression or activation of the system's components. •The dynamic simulations occur in terms of “computational” time-steps.	Simulation of GRNs (e.g., differentiation, normal-malignant transition).
Conventional continuous	•Useful for modeling biochemical reaction systems. •Output data is comparable to experimental quantitative information (e.g., signaling pathways activation or proportions of cellular populations). •Model dynamics can be simulated and interpreted in terms of real time units.	•Demands high mathematical knowledge for the proper construction and simulation of an equation system. •Requires sufficient kinetic and mechanistic details (e.g., synthesis and degradation rates). •Computationally heavy as more features and components are incorporated. •The resultant models and the hypothesis derived from them, are tightly specific to the system from which the kinetic parameters are derived	Biochemical reaction systems.
Continuous fuzzy logic	•Do not require profound kinetic and mechanistic knowledge, but allows the incorporation of quantitative information to implement a hierarchy of characteristic expression times among the network components. •The components of the system can have a continuous range of values. •Useful to simulate large biological systems that include signaling or regulatory sub-networks with scarce kinetic data available.	•The value taken by each component ranges between 1 and 0, which would relate it more to a degree of activation or expression, more than to a real concentration. •As with Boolean modeling, the accuracy of fuzzy logic models is limited by the availability of kinetic and mechanistic information.	Graded signals linked to a GRN (e.g., cytokines influencing cellular fates) influencing gene regulatory networks.

## 2. Discrete Modeling of Lymphoid Differentiation Landscape

### 2.1. Boolean Interpretation of Molecular Data

To deeply understand the gene regulatory processes involved in cellular development, C. H. Waddington introduced in 1957 the metaphoric concept of epigenetic landscape (18). He proposed a unique perspective of cellular development as a ball rolling down within a landscape formed by peaks and valleys. Following its trajectory, the ball may finally fall into a valley, representing its final position that defines a steady-state -and a cellular fate-, also known as attractor. Waddington's epigenetic landscape was formalized, among others, by S. A. Kauffman, who studied the behavior of large networks of randomly interconnected binary “genes” with a dichotomous (on-off) behavior, establishing the principles of Boolean modeling (19). The assumption of a discrete transcriptional regulation was further investigated in Drosophila embryogenesis, showing that the gradient of Bicoid morphogen resulted from averaging binary states of transcriptional activity, active or inactive, at individual nuclei level (20).

The general system's behavior and the number of attractors of a Boolean or multi-valued regulatory network depends on topological characteristics, such as the number of components and the degree of interconnectivity among them. It is now recognized that biological networks are scale-free systems, which means that the nodes have a high diversity of number of edges, including few elements with many links and many elements with few links (21, 22). Scale-freeness provides, among other attributes: network robustness, better information spreading performance, and the property that the number of attractors is almost independent from the number of nodes (23, 24).

Mathematical modeling based on Boolean regulatory networks (BRN) provides meaningful qualitative information on the basic topology of relations that determine alternative cell fates and may be used for the analysis of biological circuits without requiring explicit values of the network parameters. In this type of approach, the network nodes represent genes, transcription factors, proteins mediating signaling cascades, RNA, environmental factors, etc., and links representing positive or negative regulation between pairs of nodes. The state variable of each node takes a discrete value of 0 (inhibited, or inactive) or 1 (expressed or active) (1). The state of each node at time *t* + 1 is specified by a dynamic mapping that depends on the state of its regulators at a previous time *t*:

(1) $$\begin{array}{r} q_{k}\left(t+1\right)=F_{k}\left(q_{1}\left(t\right),\ldots ,q_{n}\left(t\right)\right) \end{array}$$

where *F*_*k*_ is a discrete function representing a logical proposition, also known as Boolean rules, constituted by elementary terms related by the logical connectives: AND (∧), OR (∨), and NOT (¬). Logical propositions satisfy Boolean's axiomatics, which complies associativity, commutativity, distributivity, absorptivity, and identity. The discrete nature of the truth values involved in Boolean logic propositions implies that this approximation is not always enough to investigate the enormous variability inherent to biological processes.

The dynamics induced by the Boolean mapping is completely determined once a set of initial expression values of the network components is specified. From a given initial set, the network nodes iteratively update their value based on the Boolean transfer rules until eventually reaching a steady-state determined by condition *q*_*k*_(*t* + 1) = *q*_*k*_(*t*). This latter condition specifies a fixed-point attractor. Then, the dynamics of a model is evaluated by tracking the trajectories from all the possible initial configurations in the states space toward the attractors. The size of the states space of a model is given by Ω= 2^*n*^ where *n* is the number of nodes in the network. Alternatively, a cyclic attractor associated to the condition *q*_*k*_(*t* + *N*) = *q*_*k*_(*t*) may also arise after the simulation of some regulatory networks, where the integer number N signals the period of the attractor. Cyclic attractors are generally interpreted as oscillatory behaviors and are sustained by at least one negative feedback circuit in the network topology, which involves an *odd* number of inhibitory interactions (25). This type of attractors can be directly associated to biological events, for example, in models predicting cell cycle oscillations (26–28) or, sometimes they can be interpreted as intermediate or oscillatory activations in multi-valued and Boolean differentiation models, as has been reported with T cell attractors (7, 29). Each fixed-point and cyclic attractor is reached from a number ω of different initial conditions. The parameter ω denotes the size of the attraction basin which may be visualized as a ratio of areas in the epigenetic landscape. Consequently, the probability that a steady state is expressed is given by *p* = ω/Ω.

**Figure 1:**
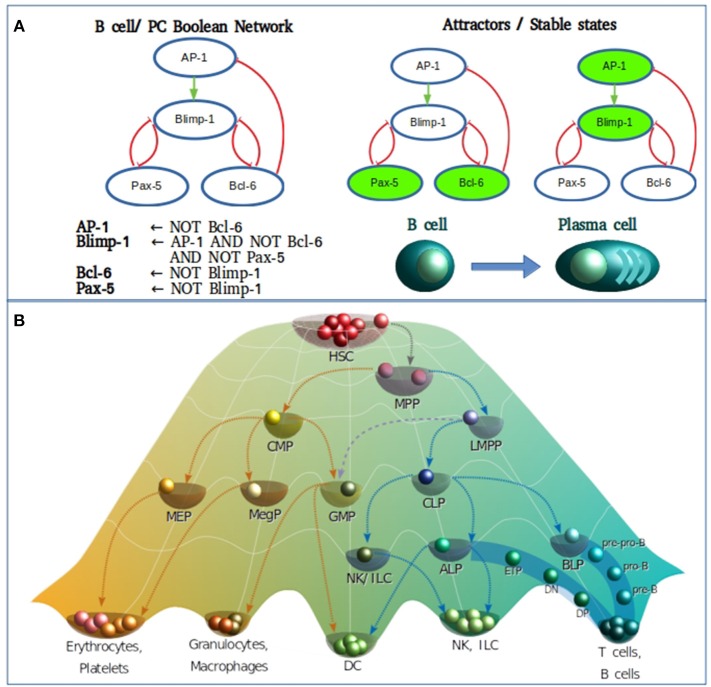
**(A)** Boolean modeling of the transcriptional core regulating naive B cell to plasma cell (PC) differentiation. Inhibitory and activation interactions are represented in the network with truncated red lines and green arrows, respectively. **(B)** Epigenetic landscape of hematopoietic differentiation where valleys represent stable cellular states, however other cellular phenotypes may be represented as transitory stages. HSC, hematopoietic stem cell; MPP, multipotent progenitor; CMP, common myeloid progenitor; LMPP, lymphoid-primed multipotent progenitor; MEP, megakaryocyte/erythroid progenitor; MegP, unipotent megakaryocyte progenitor; GMP, granulocyte/macrophage progenitor; CLP, common lymphoid progenitor; NK/ILC, natural killer/innate lymphoid cell; ALP, all-lymphoid progenitors; BLP, biased lymphoid progenitors; ETP, early thymic progenitor; DN, double negative; DP, double-positive.

To briefly exemplify how a Boolean model is constructed we used the information compilated by Bhattacharya et al. (30) of the transcriptional core orchestrating the terminal differentiation of B cells into antibody-secreting plasma cells upon antigenic stimulation. The transcription factors to be considered were Pax-5, Bcl-6, and Blimp-1. Construction of the gene regulatory network and the Boolean transfer rules are based on evidence showing the existence of a mutual repression by Bcl-6 and Blimp-1, as do Blimp-1 and Pax-5, establishing a system with two double-negative feedback loops. Pax-5 and Bcl-6 are two transcriptional factors of high expression in B cells, down-regulated by Blimp-1 after its AP-1 mediated activation. In turn, AP-1 is phosphorylated downstream B cell stimulation with lipopolysaccharides. Beside the direct inhibition of Blimp-1, Bcl-6 can also act as a passive repressor through its binding to AP-1, blocking its transcriptional activity (Figure 1A). Such information is sufficient to predict two fixed-point attractors interpretable as B-cell and plasma cell configurations. The presence of at least one positive loop containing an even number of inhibitory regulations is necessary for the generation of multiple steady states (25). This type of models has been useful to merge independently published data from different molecular circuits involved in cellular specification, to probe how these circuits orchestrates differentiation, and to generate new testable hypothesis on missing interactions or cellular transitions.

### 2.2. Genetic Regulatory Networks Underlying Lymphoid Specification

As of the discovery of HSCs by Ernest A. McCulloch and James E. Till in the 1960s, the hematopoietic system has served as the most recurrent biological model for the study of stem cell biology and differentiation. For many years, the differentiation process was represented as a hierarchical dichotomic model of strict myeloid/lymphoid branching. However, multiple observations mostly based on single cell experiments have challenged this classical view and introduced cell differentiation as a process of continuous transitions directed by two events running in parallel: the gradual commitment through the acquisition of lineage-specific features and the gradual lost of potential to generate cells of a different lineage (31–36) (Figure 1B).

In the metaphorical Waddington's view, the cell type positioned in the “summit” of the hematopoietic epigenetic landscape is the hematopoietic stem cell (HSCs) population, which resides in specialized niches within the bone marrow. Early specification begins upon “ball rolling” from the HSCs to the multipotent progenitor (MPP) attractors, either committing to myeloid or lymphoid lineages by differentiating into common myeloid progenitors (CMPs) or lymphoid-primed multipotent progenitors (LMPPs), respectively (37–39). As more is deciphered on the transcriptional network underlying the lymphoid differentiation, more is discovered about intermediate steps and novel transitional cell subpopulations. It is now well-known that LMPPs contain a mixture of myeloid and lymphoid-restricted progenitors, including early lymphoid progenitors (ELPs), giving rise to common lymphoid progenitors (CLPs), endowed with the ability of generating all types of adaptive and innate lymphocytes without noticeable myeloid potential, and some categories of dendritic cells (DCs) (40–50). The CLP population bisects into all-lymphoid progenitors (ALPs) and B-cell-biased lymphoid progenitors (BLPs) (44) that predominantly generate T and B lymphocyte precursors, respectively. From the ALP pool, some circulating progenitors reach the thymus and differentiate into early thymic progenitors (ETPs), progress to CD4/CD8 double-negative 2 cells (DN2) and DN3 cells. CD4/CD8 double-positive (DP) cells are then produced before differentiation toward CD4 or CD8 single-positive (SP) T effector cells (51). B cells reach also a partial maturation in the bone marrow (BM), following a series of sequential differentiation steps from prepro-B, pro-B, early pre-B and pre-B stages, where the rearrangement of immunoglobulin heavy-chain (IgH) genes takes place and results in the expression of the pre-B-cell receptor (pre-BCR). Downstream the pre-BCR activation and the signaling cascade deriving in a clonal expansion and the subsequent cell cycle arrest, a second wave of recombinases Rag1 and Rag2 expression induces the rearrangement of the immunoglobulin light-chain (IgL), marking the transition from pre-B-cell to immature B cells (44, 52, 53). Upon migration to the secondary lymphoid organs, T and B lymphocytes are exposed to antigens and signals provided by a number of immune cells in the microenvironment.

**Figure 2:**
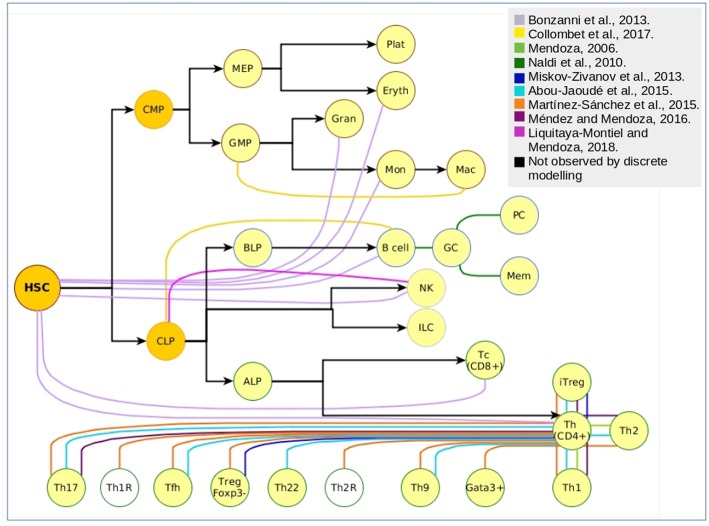
Cellular attractors and transitions from the hematopoietic landscape reproduced through discrete modeling. Each color represents an independent article of hematopoietic discrete modeling, black arrows represent the direction of hematopoiesis toward the myeloid and lymphoid lineage. White nodes represent predicted phenotypes that have not been associated to experimental findings.

Even though differentiation transitions are now recognized as continuous processes, commitment to stable phenotypes is dependent on molecular switches that act as lineage-determining steps, what has made the differentiation process a target for its simulation through discrete models. More specifically, hematopoietic differentiation has been approached with discrete models at many levels (Figure 2), from the top of the epigenetic landscape hill sloping down to the final stages of mature cells production. The main type of information provided by the construction and simulation of BRN is obtained after the confirmation of the functional integration of the proposed components. This generally occurs validating the attractors and transitions with previous experimental observations. To compare with experimental data, Boolean models are subjected to different types of perturbations including permanent mutations (e.g., gene knock-out or overexpression), or temporal changes in the nodes activation value which can be understood as triggering cues for network state transitions. An example of this type of evaluations is the case of the hematopoietic stem/progenitor (HSPC) network model generated by Bonzanni. The HSPC model contains ten genes expressed in the immature stem cell population besides GATA1, which is expressed in the early progenitor MPP (2). The dynamic simulation of the HSPC network generated two single state attractors, one with an erythroid cell profile, and one with a non-hematopoietic cell profile with all genes turned-off, as well as a periodic attractor composed of 32 interconnected states with oscillatory activation values for four genes (Gata2, Zfpm1, Erg, and Eto2a) compatible with single cell gene expression data from HSPCs (54). The activation state of one or more genes in the states comprising the HSPC complex attractor were modified to compute the dynamic transitions and mapping the developmental route from HSPC toward erythrocyte, granulocyte, monocyte, natural killer (NK), B cell, CD4, or CD8 T cells profiles (55). This type of evaluation provided information about the stability of the HSPC attractor, the type of genes involved in the developmental route considering those that trigger differentiation, and the suggestion that there were missing interactions or components that avoid differentiation reversal.

Furthermore, the myeloid/lymphoid branching has been addressed through the assembly of a GRN integrating 23 nodes that, when computed using a logical multi-valued formalism, produced four stable stages corresponding to CLPs, B-lineage cells, granulocyte-monocyte progenitors (GMPs) and macrophages (9). As previously discussed, even the network assembling may constitute a useful mechanism to propose novel interactions. This was the case with this model, by envisioning three missing regulations: negative regulation of C/EBP(α) transcription by Foxo1, E2A activation by Ikaros, and Gfi1 positive regulation by Pax-5. Moreover, the model was useful to explore molecular mechanisms of transient induction of the transcription factor C/EBP that down-regulates the transcriptional core of B cell specification and promotes an irreversible trans-differentiation toward macrophages. The theoretical findings complemented the results of a previous experimental report where B cells were transdifferentiated into macrophages by the enforced expression of C/EBP and C/EBP, but without a full understanding on the molecular steps leading to the loss of early and late lymphoid markers and acquisition of myeloid-specific genes (56). Predictions from these models and their perturbations might be useful to unravel the pathobiology of diseases where neoplastic cells concomitantly express myeloid and lymphoid markers (57–61). Also, early branching models may help to deepen the research on plasticity-related processes, such as those suggested to be involved on leukemia lineage switching and relapse (62, 63). It has become of particular interest the integration of microenvironmental cues capable of influencing and regulating transcriptional cores, particularly to approach the two-way feedback between cells and their surrounding microenvironment.

### 2.3. Microenvironmental Modulation of Lymphoid Differentiation and Plasticity

The applicability of discrete models seems to be simplistic but their scopes are expanding in parallel with the knowledge on cellular heterogeneity and plasticity. Their flexibility for the analysis of biological systems integrated with multiple types of molecular events makes them a useful tool for evaluation of different microenvironments that consider the modulation of genetic and signaling networks. Molecules, such as integrin, cytokine, or antigen receptors, might be included in computational models as they are involved in maintaining particular hematopoietic compartments, enhancing proliferation, regulating apoptosis or migration, or guiding differentiation to either one phenotype or another. As previously mentioned, some of these processes become discrete cellular decisions with a bi-modal behavior as a result of the combined effect of their connectivity in molecular networks and noise (64–66).

Early logical mathematical approaches for modeling lymphocyte behavior upon antigen exposure preceded the development of networks that connected intracellular events regulating the cellular fates of hematopoietic progenitors and lymphocytes (67, 68). However, as the different subtypes of lymphocytes were discovered, efforts focused on the reconstruction of the GRN underlying the emergence of mature phenotypes in response to variable microenvironmental factors under normal and pathological conditions. The first model of lymphoid differentiation branching using a discrete perspective resulted from the transformation of a previous continuous model based on Hill functions describing the polarization of naive Th cells (Th0) into Th1 or Th2 cells (69). The Boolean version proposed by Mendoza (3) integrated 17 nodes and replaced the transcription factor Gata-3 positive self-feedback loop in Yates' model (70) with a more refined functional feedback circuit engaging Gata-3 and interleukin-4 (IL-4) (71). The activation of this functional circuit characterizes the Th2 cell subtype (72, 73). Besides recovering the Th0 polarization into Th1 and Th2, the model was able to describe the transition between Th1 and Th2 attractors by the stimulation with IFN, IL-4, or the combination of IL-12 and IL-18. Later on, the model was extended to include novel transcription factors, cytokines, and signal transduction molecules to describe additional fates to T regulatory (Treg) and Th17 cells (29). More refined molecular data has resulted in the reconstruction of larger versions and their simulations, predicting a larger repertoire of Th cell subsets including Tfh, Th9, Treg, iTreg, Th9, Th17, Th22, and T regulatory Foxp3 independent cells (6, 7, 74).

B and NK cells have been less studied by mathematical modeling. During terminal B cell differentiation in the germinal centers of secondary lymphoid organs, the exposure to particular environmental factors, including the antigen-mediated activation of the B-cell receptor (BCR), defines the transition of the naïve B cell to a memory cell or an antibody-producing plasma cell. This terminal differentiation of B cells has been simulated as a Boolean model that recovered four cellular profiles: naive B cell before and after arriving to the germinal center (GC), memory cell (MC) and plasma cell (PC) (8). The B cell model reproduces not only the expected cellular attractors, but also the transitions with biological significance. Of note, it predicts four interactions that have not been declared experimentally but are suggested through indirect mechanisms: two self-feedback loops involved in Pax5 and Bcl6 activation, the positive regulation of Bcl6 by Pax5, and the inhibition of Pax5 by Irf4.

On the other hand, NK cell biology has been recently approached by a Boolean model providing a CLP attractor that transits toward pro-B, early T progenitor, or three different subtypes of NK attractors, depending on the activation pattern of IL-7, IL-15, and Delta ligand (75). NK cell subsets are characterized by differential expression of the transcription factors T-bet and Eomes. The NK attractor reached after CLP is stimulated with IL-15 activates both transcription factors and correlates with highly cytotoxic NKs both, in humans and mice periphery. On the other hand, perturbation of the CLP attractor with combined activation of IL-15/IL-7 or IL-15/Delta ligand, leads to a T-bet- Eomes^+^ profile correlating with BM NKs or T-bet^+^ Eomes^−^ compatible with liver NKs (76). The incorporation of more transcriptional regulators may lead to new hypothesis about the branching step between NK cells and the more recently described, innate lymphoid cells (ILCs). It has been purposed that CLPs transition to NK lineage may have an intermediate step of a common progenitor for NKs and ILCs with a probable expression of transcription factors shared by both lineages, such as Nfil3 and TOX (77). In contrast to adaptive lymphocytes, the knowledge on transcriptional circuits controlling ILC development remains limited, although their role in the orchestration of immune responses has become of particular interest. ILCs are enriched in mucosal tissues and have been correlated with the progression of allergic, gastrointestinal, and central nervous system inflammatory diseases, like inflammatory bowel disease (IBD) and multiple sclerosis (78, 79). Similar to T lymphocytes, ILCs show plasticity under microenvironmental challenges modifying their cytokine secretion patterns and in consequence, the response exerted by other cells of the immune and adaptive branches (80).

The continuous integration of data, an inevitable process to improve computational modeling of biological systems, leads to the generation of large and complicated networks. To facilitate their analysis, large networks can be subjected to model reduction, a process of iterative removal of particular nodes and redirection of the logical rules that ideally, preserve the reachability of the attractors while keeping the main dynamical properties (29, 81, 82). Model reduction considers that, in a number of cases, a central core of nodes drives the dynamics of other dependent nodes. One of the simplest methodologies to drive model reduction (16) consists in excluding from the steady-state computation those nodes that follow linear downstream pathways. For example, the consecutive rules *q*_3_(*t* + 1) = *q*_2_(*t*) and *q*_2_(*t* + 1) = *not q*_1_(*t*) may be transformed into *q*_3_ = *q*_2_ = *not q*_1_, so that the state of *q*_1_ automatically determines *q*_2_ and *q*_3_. A more elaborate example would be *q*_5_(*t* + 1) = *q*_4_(*t*) *and* [*q*_4_(*t*) *or q*_3_(*t*) *or not q*_2_(*t*)] which leads to *q*_5_ = *q*_4_ *and* [*q*_4_ *or q*_3_ *or not q*_2_]; using the Boolean absorption rule *a and* (*a or b*) = *a*, this expression is finally transformed to *q*_5_ = *q*_4_. In this latter case, the steady state of *q*_5_ is merely determined by *q*_4_, independently of the state of *q*_3_ and *q*_2_ which appear in the original rule. Furthermore, model reduction is a useful tool to identify regulatory cores or redundant signal transduction pathways, reduce the states space and obtain qualitative data comparable to experimental results (83–85). An alternative to deal with networks whose size complicates the exhaustive analysis of their state space, consists in the evaluation of cellular transitions assessed with a computational technique known as model checking (6). Model checkers are based on the transformation of states space into graphic or symbolic structures that facilitate verification of properties and trajectories, allowing fate mapping of all possible cell transitions and emerging as a potent predictive tool for cellular plasticity under multiple microenvironmental contexts.

The role of the microenvironment in lymphoid differentiation is successfully implemented in the reviewed models by considering the hypothesis that cytokines are either absent or present, and do not care about graded availability. Other models integrate assumptions to simulate signal processing and propagation using a discrete model, such as the models of the downstream events occurring after the activation of the T-cell receptor (TCR) (11, 12). Saez-Rodriguez et al., based a Boolean model in a large network of 94 nodes and considered that some signaling events occur in a different timescale, so that logical rules were updated in a first and second wave depending on the molecular nature of the event. From the attractors resulting after the simulation, the authors made predictions about the signaling cascade activated by the receptor engagement and confirmed them experimentally. The implementation of two updating waves is a way to recognize that the cellular events occur in different timescales, for example biochemical reactions occurring in the cytoplasm (e.g., molecular inhibition by phosphorylation) are faster than the transcriptional modulations (e.g., transcription factor translocating to the nucleus and binding to a gene promoter that will be activated). Even though their utility, it is necessary to recognize that Boolean models are sometimes insufficient, particularly when there is enough data about the continuous concentration of a biomolecule determinant for the process that is being modeled.

The study of chronic diseases has strongly influenced the understanding of how slight changes derive in the complete perturbation of complex biological systems. If it were desired to simulate the way in which the progressive accumulation of pro-inflammatory factors in the intestinal tract perturb the proportions of T cell populations, the use of Boolean models would be of very limited use to investigate the transitory stages between the healthy attractor and a pathological attractor, like in IBD (78).

## 3. Modeling of Continuous Variables to Study Lymphocyte Diversity

Modeling lymphoid cells production or activation may require the integration of molecules involved in dosage-dependent effects, as is the case of ligand-receptor affinities, cytokine gradients and even some transcription factors like C/EBP and PU.1 (9, 56). As suggested by the number of publications, continuous mathematical models are the most recurrent tool for the study of lymphocyte development and response and are useful tools to evaluate population dynamics and receptor repertoire (86–89).

However, most time parameters are fitted to experimental data without a deep understanding of molecular mechanisms, unless enough kinetic and biochemical information is available. Some cellular processes involving dosage variations may still be simulated with discrete approaches using multi-valued models or probabilistic Boolean networks, but there exist other alternatives to integrate discrete and continuous molecular events like the construction of hybrid and fuzzy models. On one side, hybrid models have been applied to simulate the activation of Th and B lymphocytes by DCs, and their subsequent departure from the lymph node. The cellular entities and the replication steps were modeled in terms of discrete variables, while the migration was simulated by means of differential equations involving continuous variables and parameters (e.g., chemokines concentration and diffusion, cellular velocity) (90). On the other side, Boolean models may be transformed to continuous systems using fuzzy logic (5, 8, 15–17, 91, 92). These approaches may be useful to use existing GRN of lymphoid differentiation and activation to model complex scenarios that involve intercellular communication among immune cells, interaction of immune cells with normal or pathologic tissue, and immune cell population transitions in response to microenvironment remodeling.

### 3.1. Dosage Variations in Multi-Valued and Probabilistic Models

The molecular pathways participating in TCR signaling have been successfully modeled with a set of differential equations. The first step for T lymhpocytes activation involves a process known as ligand discrimination that differentiates between weak and strong binding antigens. After TCR engages with peptides processed and expressed on the surface of antigen-presenting cells, a well-regulated discrimination between self and non-self antigens is triggered. The simulation of TCR activation as a continuous model suggested that the MAPK cascade is the responsible for this discriminatory engagement process. A negative feedback loop that modulates the TCR response until an ERK activation threshold is reached may take place, resembling a bimodal behavior (93). The model was expanded to answer the question of how stochastic variations of protein expressions among a clonal population of CD8 T cells could affect their responsiveness. Variations on the expression of CD8 and two components of the MAPK signaling pathway, ERK-1 and SHP-1, generate dispersion in responsiveness among individual cells, but the co-regulation of CD8 and SHP-1 restrain the phenotypic variability (94). It was later discovered that the ligand discrimination process influences T cell differentiation to Treg or Th phenotypes through the downstream modulation of PTEN and Akt/mTOR signaling pathways (95, 96). To represent a weak or strong ligand affinity, a multi-valued model was useful allowing three possible activation levels of TCR and PI3K nodes (off = 0, low = 1, and high = 2). The computational simulations of the model corroborated that low TCR signal favors Treg differentiation, while a stronger signal result in the induction of Th profile (97). Additionally, varying the number of rounds or time-steps for TCR activation, as an approach for ligand binding lifetimes, showed that the Th phenotype is more rapidly stabilized than a Treg profile, suggesting that the transition from naïve to Treg cells is less direct than the Th differentiation. The generation of Treg cells goes through intermediate stages during which the secretion of IL-12 is promoted and activates the PTEN signaling pathway that enables Foxp3 permanent activation (97). Under high TCR signaling, Foxp3 is transiently activated but further turned off by mTOR pathway, while the Akt-dependent regulation of T cell fate choice is also dependent on the differential phosphorylation of additional proteins (98). There are ongoing studies focused on the blockage of TCR signaling by some pathogens like *Yersinia pseudotuberculosis* (99).

To deepen in the composition of the microenvironmental patterns affecting the diversity of T lymphocytes, a probabilistic Boolean control network (PBCN) was developed for simulation of all possible microenvironments combining nine external signals including TCR activation, TGF-β and IFNγ cytokines, and six interleukines. In contrast with conventional Boolean models, PBCNs contemplate activation probabilities as an approach to input dosages, increasing the range of testable microenvironments (74). Experimental research on T lymphocytes diversity has led to the discovery of intermediate phenotypes that co-express lineage-specific transcription factors from more than one T cell subset, such as Th1-Th2 and Th1-Th17 cells identified on bacterial and parasitic infection (100–102). Through a sensitivity analysis of the PBCN the minimum microenvironment requirements have been identified, on composition and dosage, for the description of each of the 10 T cell profiles. In addition, they have been used to predict the way in which different input patterns influence the internal balance determining the phenotype of canonical and complex cellular profiles, such as cells with mixed phenotypes. With a continuous model constructed to simulate iTreg-Th17 differentiation, Hong and collaborators reported a double expressing phenotype with either regulatory or dual (regulatory and proinflammatory) functions *in vivo*. This mixed phenotype is suggested to be a stable state reached from the transition of single-expressing cells, iTreg and Th17. Th17 and iTreg cells are able to produce TGF- which may either increase the percentage of both types of cells, or induce the transition from single-expressing to double-expressing cells. The iTreg-Th17 model was also used to analyze how different concentrations of TGF- influence the rate of co-existing cellular subtypes, making evident that priming factors not only drive differentiation events, but also promote cell heterogeneity (103).

The models presented in this section have different limitations. The multi-level and probabilistic models do not allow the integration of temporal hierarchies in the events involved in the biological system of interest, particularly important when modeling more than one type of cellular processes. The continuous model includes a limited number of components depending on the availability of kinetic parameters or enough information to establish assumptions. As an alternative, fuzzy logic can merge large transcriptional regulatory networks participating in cell differentiation and plasticity, with qualitative knowledge about the kinetics of signaling pathways involved in the transduction of microenvironmental variations, for example, events proceeding relatively faster than others, or ligands binding to receptor above other ligands.

### 3.2. Continuous Simulation of Discrete Differentiation Networks

Extracellular signals and some intracellular components are continuous variables and their adequate representation in mathematical models may determine the simulation of lymphoid cellular fates like differentiation, phenotypic transitions and activation. The transformation of discrete models to a set of differential equations is useful to identify additional attractors and unstable states with biological relevance. In a comparison between Boolean and continuous simulation of a B cell terminal differentiation network, the continuous counterpart provided three additional stable states with intermediate values of Bcl-6 and/or Irf4; however, only one of them was comparable with a previous reported phenotype that may correspond to the centrocytes found during the germinal center selection (8, 104). This intermediate phenotype together with centroblasts, are particularly important in the study of follicular lymphomas characterized by an accumulation of cells unable to reach terminal differentiation stages.

In comparison with Boolean models, the computational simulation of continuous fuzzy models is simpler and in consequence faster, thus allowing the integration of independently developed BRN without caring a lot about the number of resultant equations. An example is the T/B lymphoid differentiation model of 81 equations representing cytokines and transcription factors that lead to ten attractors with Th0, Th1, Th2, Th17, Treg, cytotoxic T lymphocyte, DP T lymphocytes, CD8 T naive, naive B cell, and PC profiles (105). The attractors obtained by the continuous model show a higher compatibility with experimental data than previous discrete models. In this case, all the attractors display intermediate values for Ikaros, Gfi1, and PU1. For each of the three transcription factors there exists strong evidence that associates this intermediate expression with the delimitation toward lymphoid lineage during hematopoietic differentiation (106–108). The intermediate modulation of PU1 and Ikaros was also reproduced with a different continuous model of B-lymphocyte lineage commitment, evidencing their participation in the transcriptional core that reproduces the irreversible transition from LMPP to lineage restricted progenitors expressing IL-7R (109).

An additional application of fuzzy logic models is the simulation of virtual cultures where independent GRNs, representing multiple cells, may interact with a microenvironment expressing graded and dynamic concentrations of cytokines. A virtual culture of T lymphocytes was proposed by Mendoza to evaluate the evolution of 100 cells with an initial Th0 configuration after being stimulated with IFN, I-4, TGF alone, or TGF in combination with IL-6. The phenotype of each cell was determined by the activation state of each of the 36 nodes integrating the internal Th differentiation network, in turn, regulated by 11 cytokines produced depending on each cellular profile (Th0, Treg, Th1, Th2, and Th17). The produced cytokines involved endocrine and paracrine signaling to evaluate the final balance of the T lymphocyte subpopulations arising from different types of stimulus (91). This particular implementation is computationally expensive, but represents a more realistic approach to analyze the interaction between heterogeneous populations of immune cells susceptible to transit among phenotypes, including dynamic secretion patterns that influence the composition of the microenvironment.

### 3.3. From Discrete to Continuous Using Fuzzy Logic

A more realistic approach must considerate that the expression levels, concentrations, and parameters of biological systems may take any value within a continuous range limited only by functionality constraints. In this case, the discrete dynamic mapping given by Equation (1) may be generalized by introducing a set of ordinary differential equations (ODEs) for the rate of change of the expression level of the network components. For *k*-th node, this is written as

(2) $$\begin{array}{r} \frac{dq_{k}}{dt}=\mu \left[w_{k}\left(q_{1},\ldots ,q_{n}\right)\right]-\alpha_{k}q_{k}. \end{array}$$

Here, μ[*w*_*k*_] is an input function that expresses a continuous realization of the Boolean rule *w*_*k*_ (see below), while α_*k*_ is a decay rate. In this scheme, the equilibrium states of the system are defined by the steady-state condition *dq*_*k*_/*dt* = 0, which leads to

(3) $$\begin{array}{r} q_{k}^{s}=\frac{1}{\alpha_{k}}\;\mu \left[w_{k}\left(q_{1}^{s},\ldots ,q_{n}^{s}\right)\right], \end{array}$$

where the superindex *s* denotes the steady-state value. A straightforward consequence of this is that the expression level of node *k* is strongly dependent on its decay rate. In the case α_*k*_ > 1, a node will be under-expressed with respect to the value attained for α_*k*_ = 1; in particular, for α_*k*_ ≫ 1, the expression of that node will be completely inhibited: $q_{k}^{s}\to 0$. The converse also holds: if α_*k*_ < 1, a node will be relatively over-expressed [it must be noticed that a decay rate α_*k*_ < 1 may lead to a steady expression value *q*_*k*_ > 1. Although in fuzzy logic the values of the variables are assumed to be constrained to the interval 0 ≤ *q*_*k*_ ≤ 1, values >1 are not excluded by the formalism, and it is a matter of convenience the range in which the variables are defined (110)]. It follows that modifications of the characteristic decay rates of network components may alter the steady expression patterns arising from the nodes interactions. This may be interpreted as a modulation of the metaphorical or Waddington's epigenetic landscape which eventually may lead to transitions between attractors associated to different cell fates. This approach has been formerly employed, for example, to model plastic phenotype changes in T CD4^+^ lymphocytes (92).

The translation of the interactive Boolean rules to the continuous domain may be accomplished by considering an approach based on fuzzy logic. Fuzzy logic is a theory aimed to provide formal foundation to approximate reasoning with applications in physical, biomedical, and behavioral sciences. It is characterized by a graded approach (110–112), so that the degree to which an object exhibits a given property is specified by a membership (or characteristic) function μ[*w*_*k*_], with truth values ranging from total falsity, μ[*w*_*k*_] = 0, to totally true, μ[*w*_*k*_] = 1. For example, the property of “being a good person” implies that there is a set of persons that share certain characteristics with no definite boundary. Fuzzy logic satisfies an axiomatic similar to the implied in Boolean logic, except for the identity principle, meaning that the principle of no-contradiction does not hold. Thus, although seemingly paradoxical, a proposition *w* and its negation 1 − *w* may be simultaneously true. For example, the assertion “he was not a good, but not bad guy” has a meaning in language theory. In biological systems, fuzzy propositions may describe cases in which a cell displays an intermediate expression pattern that does not necessarily belong to a specific phenotype. That is the case of individuals with food allergies, in which Treg cells produce IL-4, which is a characteristic usually ascribed to Th2 cells. Similarly, diseases like rheumatoid arthritis or colorectal cancer are associated to the expression of IL-17^+^Foxp3^+^ Treg cells or RORγ*t* + Foxp3^+^ Treg cells, respectively. The absence of no-contradiction is formally expressed by the equation *w* = 1 − *w*, with solution *w* = 1/2. It follows that the value *w* ≡ *w*_*thr*_ = 1/2 may be interpreted as a threshold between falsity and truth.

Similar to the Boolean approach, in the continuous regime the network regulatory interactions are characterized by fuzzy logic propositions denoted here as *w*_*k*_[*q*_1_(*t*), …, *q*_*n*_(*t*)]. They are either inferred from experimental observations or suggested by inner consistency requirements. In fact, a translation scheme from the discrete to the continuous scenario may be straightforwardly implemented translation by replacing the Boolean connectives AND, OR, and NOT, for its fuzzy counterparts. In fact, the definition of fuzzy connectives is not unique, and a number of different alternatives not entirely equivalent, have been proposed. In the following table we present Zadeh's original proposal (111) and a probabilistic-like scheme (110):

**Table:** 

Boolean	Zadeh	'Probabilistic'
*q* AND *p*	min [*q, p*]	*q*·*p*
*q* OR *p*	max [*q, p*]	*q*+*p* − *q* · *p*
NOT *p*	1 − *p*	1 − *p*

Both schemes satisfy the modified Boolean axiomatics discussed above. However, the probabilistic-like scheme leads to continuously differentiable expressions if q and p are differentiable. This is a desirable condition when dealing with ODEs systems. Furthermore, it shows the same properties as joint probabilistic distributions for independent variables, so that probabilistic statements may be directly translated into fuzzy propositions.

An example of translation from the Boolean to the fuzzy framework is

$$\begin{array}{l} W\left[p,q,r\right]=\left(q\vee p\right)\wedge \neg \;r\;\to \;w\left[p,q,r\right]\\ \;=\left(q+p-q\cdot p\right)\cdot \left(1-r\right). \end{array}$$

Continuous logical propositions can be used to construct an explicit expression of the characteristic function μ[*w*_*k*_]. In the discrete Boolean approach, this function would be equivalent to a step Θ function:

$$\mu [w_{k}]\to \Theta \;[w_{k}-1/2]=\{\begin{array}{cc} 0 & \text{if }w_{k}\;<\text{1/2};\\ 1 & \text{if }w_{k}>\text{1/2}. \end{array}$$

In the continuous approach this behavior may be approximated by a characteristic function with a sigmoid structure that gradually changes from a null to a unit value. Many functions share this property. An expression employed in a number of applications of fuzzy logic in systems biology is the logistic function:

(4) $$\begin{array}{r} \mu \left[w_{k}\right]=\frac{1}{1+\exp\left[-\beta \left(w_{k}\left(q_{1},\ldots ,q_{n}\right)-w_{thr}\right)\right]} \end{array}$$

**Figure 3:**
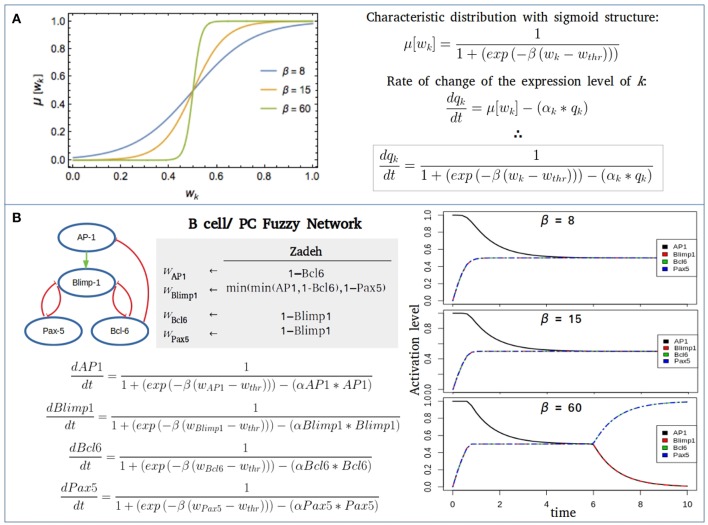
Fuzzy networks. **(A)** Characteristic distribution $\mu \left[w_{k}-w^{thr}\right]$ for a threshold value *w*^*thr*^ = 1/2 of the logical proposition *w*_*k*_, and different values of the saturation parameter β. In the case β ≫ 1, the characteristic distribution becomes a step-like distribution. **(B)** Fuzzy modeling of the transcriptional core regulating B cell to plasma cell (PC) differentiation using three different saturation values (β = 8, 15, 60), *w*^*thr*^ = 1/2 and the decay rate for each component α = 1. For the initial state of network all nodes were considered inactive, except AP-1, the promoter of the PC differentiation. When β = 60, a full B cell attractor is reached with no final expression of AP-1 or Blimp-1.

Here, *w*_*thr*_ is a threshold value such that if *w*_*k*_ > *w*_*thr*_, then *w*_*k*_ tends to be true (or expressed). Usually *w*_*thr*_ = 1/2. The parameter β is a saturation rate that measures the pace of the transit from an unexpressed to an expressed state, as displayed in Figure 3. We observe in the figure that this pace is gradual for small β, and steep for large β. In the case β ≫ 1, μ[*w*_*k*_] → Θ[*w*_*k*_ − *w*_*thr*_]. This latter result, together with the steady-state condition given by Equation(2), implies that in the case is another manifestation of the robustness of the qualitative predictions generated by the fuzzy approach. A related result is that in the limit β ≫ 1 and α_*k*_ = 1 for every network interaction, then the steady-state condition given by Equation (7), guarantees that the set of fixed-point attractors resulting in the Boolean and fuzzy approaches coincide by construction. On the contrary, the corresponding sets of periodic attractors usually differ.

It may be argued that the predictions obtained in the fuzzy formalism may depend on the specific form of the characteristic function μ[*w*_*k*_]. In fact, there are multiple expressions employed for example, in engineering applications and control theory, such as triangular, trapezoidal, or Gaussian functions (113). However, the logistic structure of μ[*w*_*k*_] considered in this review may be derived, rather than postulated, by introducing the concept of Shannon's information entropy (work in preparation). This is related with the number of independent ways in which a logical proposition may acquire partial values of truth for fixed values of the parameters β and *w*_*thr*_. In other words, the more general expression involving the least number of assumptions concerning a graded approach to truthiness of a fuzzy proposition is the logistic distribution. Interestingly, the mathematical formalism associated to fuzzy regulatory networks including the description of logical rules with a logistic structure is formally equivalent to that employed in the computation of neural circuits in the theory of neural networks (114).

Another useful (and equivalent) representation of the characteristic function may be derived by considering that the expression levels of biological variables, such as the concentrations or the affinities of a given molecule, may show variations involving several orders of magnitude. In that case, it may be convenient to introduce in the description the logarithms of the corresponding quantities. This is easily performed by means of the change of variable *w*_*k*_ = ln *x*_*k*_ and substituting this into Equation (8), leading to the well-known expression for the Hill function:

(5) $$\begin{array}{r} \tilde{\mu }\left[x_{k}\right]=\frac{x_{k}^{\beta }}{x_{thr}^{\beta }+x_{k}^{\beta }}, \end{array}$$

where the parameter *x*_*thr*_ represents the value at which $\tilde{\mu }\left[x_{k}\right]$ acquires half its maximum value. The Hill function and its negation $1-\tilde{\mu }\left[x_{k}\right]$ display both a sigmoid shape and have been widely employed in the modeling of biochemical, physiological, and pharmacological processes. A paradigmatic example is the set of non-linear differential equations

(6) $$\begin{array}{r} \frac{dx_{k}}{dt}=\frac{a_{k}x_{k}^{\beta }}{x_{thr}^{\beta }+x_{k}^{\beta }}-\frac{b_{k}x_{thr}^{\gamma }}{x_{thr}^{\gamma }+x_{k}^{\gamma }}, \end{array}$$

describing, for example, the simultaneous binding (unbinding)of β (γ) ligands to (from) a single receptor. This latter representation has been employed in the construction of a GRN that characterizes fate decisions and reprogramming signaling pathways of pancreatic cells (115). Although this latter model was not built within the fuzzy logic approach, we observe that in this and numerous instances a formal equivalence may be established by a convenient re-scaling of the variables and parameters involved in the description.

### 3.4. Self-Organization and Time Ordering

To describe the transitions between distinct steady states, in conjunction with fuzzy logic elements, general concepts of theory of non-equilibrium phase transitions and self-organization are highly relevant to consider. The adaptation of that theory to the fuzzy logic modeling scheme allows a sound description of the transitions between the different disease stages. In the description the transitions between steady states it is important to contemplate that differentiation from a multipotent stem or progenitor state to a mature cell is an essentially irreversible process, and that the associated changes in gene expression patterns exhibit time-directionality. Whereas, in equilibrium systems time-irreversibility is a direct reflection of the second law of thermodynamics, the cell's gene regulatory network represents a system far from thermodynamic equilibrium. These problems have been contemplated by the theory of cooperative phenomena, non-equilibrium phase transition and self-organization (116). Accordingly, cooperative phenomena arise from non-linear interactions of a large number of elementary subsystems (represented here by the fuzzy logic rules), leading to the emergence of organized patterns or phases. The theory relies upon two main concepts, the existence of ordering and control parameters. The order parameters are those variables that mainly drive the system organization, while the control parameters are variables whose value determines which of the possible organizations is actually realized. In the case of thermodynamic systems, an order parameter would be the density, which defines an aggregation state, such as liquid, solid, or gas. These states may somehow “compete,” in the sense that one or other may prevail depending on the value of an external control parameter, such as the temperature of the system, for fixed values of pressure and volume. In the context of fuzzy GRNs, the order parameters are the activation patterns that specify the different cell phenotypes, determined in turn, by the activation state of central nodes or functional moduli of the GRN, while the control parameters are those involved either in the logic rules, or those characterizing the decay rates {α_*k*_}. This latter set is of prime importance. Given that α_*k*_ = 1/τ_*k*_, where τ_*k*_ is a characteristic expression time, the set {α_*k*_} implicates a hierarchy for the temporal expression of the GRN components. By assuming that an ordering α_1_ > α_2_ > … can be constructed, this procedure induces an associated ordering τ_1_ < τ_2_ < …. As in the thermodynamic example, the phenotypic landscape (or state space) may be explored by varying each of the control parameters α_*k*_, while maintaining fixed the rest. As a consequence, transitions between different ordered phases may be induced by modifications of the control parameters. This is similar to the description of chemical reactions in the reaction coordinate space, where the substrate and product states are separated by an activation energy barrier; when an enzyme is added the activation barrier is lowered, and the chemical reaction ensues. In Waddington's landscape context, this mechanism may be interpreted as alterations of the peaks separating the valleys, allowing the exploration of the landscape and transit between valleys. This kind of description has been employed in the modeling of the long-term progression of type-2 diabetes based on a GRN for pancreatic beta cells. In this case, the organization patterns correspond to states identified with health, metabolic syndrome, or manifest diabetes. The alteration of decay rates of key cellular components involved in inflammatory and metabolic pathways lead the transitions between different disease stages.

An important consequence of establishing a time ordering, is that the system dynamics may discriminate among “slow” and “rapid” variables and it may be shown that the main dynamics is driven by “slow” variables, while the “rapid” variables adapt almost instantaneously to the environment defined by the “slow” ones. It turns out that the relaxation times of the order parameters are usually much longer than those of irrelevant variables and thus work like control parameters of the system. Irrelevant variables decay rapidly to a steady state, so that they may be effectively eliminated from the overall dynamics. In this view, the order parameters define the general features of the system, including the final expression patterns associated to a set of initial conditions, while less relevant variables adapt their values to the instructions dictated by the order parameters. This property may be relevant in the study of multifactorial diseases, since it could help in the identification of variables that constitute a target for the development of therapies.

Another element that may be relevant in the study of transitions between steady states is the consideration of extrinsic and intrinsic noise, i.e., the existence of random interactions inherent to every biological system. Depending on its intensity, the existence of noise may drastically alter the predictions yielded by the deterministic formalism considered before, especially at bifurcation points of the landscape, where noise may accelerate a transition rate between neighbor attractors. In the chemical reaction analogy, this is equivalent to adding heat to the process. The action of noise may be incorporated in the fuzzy logic approach by assuming that this is characterized by a stochastic variable ξ(*t*), with zero mean 〈 ξ(*t*) 〉 = 0, and statistical dispersion given by 〈 ξ(*t*) ξ(*t*′) 〉 = *DG*(*t* − *t*′). Here, *D* is a diffusion coefficient, and *G*(*t* − *t*′) is a function that characterizes the duration of the self-correlation of the variable ξ. The case in which *G* is a Dirac delta, i.e., a sharply peaked distribution only for *t* = *t*′, and null elsewhere, corresponds to a white noise with no-memory effects.

The fuzzy stochastic dynamics (16) can be described by a Langevin equation (116, 117):

(7) $$\begin{array}{r} \frac{dq_{k}}{dt}=\mu \left[w_{k}\left(q_{1},\ldots ,q_{n}\right)\right]-\alpha_{k}q_{k}+\xi_{k}\left(t\right), \end{array}$$

with steady states given by the mean value $\langle q_{k}^{s}\rangle =\langle \mu \left[w_{k}^{s}\right]\rangle /\alpha_{k}$. In the same way as in the deterministic approach, the parameters α_*k*_ control the relative heights of peaks and valleys in the *mean epigenetic landscape*. In the case of small noise (*D* ≪ 1) the time-dependent solutions are composed by the mean path $\langle q_{k}^{s}\left(t\right)\rangle$ plus random fluctuations around this path, similarly as dust particles driven by a gentle breeze. The Langevin formalism was implemented by Zhou et al. (115) by means of a GRN addressed to study the processes of differentiation and cell fate reprogramming in pancreatic cells. They show that it is possible to recapitulate the observed attractors of the exocrine and β, δ, α endocrine cells and to predict which gene perturbation can result in a desired lineage reprogramming.

A related approach rests upon a *probabilistic or quasi-potential landscape* (118, 119). In this case, it is not the ensemble of stochastic trajectories *q*_*k*_(*t*), but their probability distribution *P*[*q*_*k*_(*t*)] what constitutes the central concept. One may envisage an epigenetic landscape in which the maximal expression probabilities lie over the deepest (or wider) attraction basins, while the minimal probabilities lie over the hills' tops. Thus, the probabilistic landscape corresponds to an inverted realization of the epigenetic landscape. It can be shown that the probability distribution *P*[*q*_*k*_(*t*)] satisfies the Fokker-Planck (FP) diffusion equation (116, 117), and that the information contained in this formalism is equivalent to that inherent to the Langevin approach. It has been proposed by Wang et al. that the genetic circuitry connections in the quasi-potential landscape imposes the arrow of time in stem cell differentiation, so that the generic asymmetry of barrier heights indicates that the transition from the uncommitted multipotent state to differentiated states is inherently unidirectional.

## 4. Lymphocyte Involvement in Chronic Diseases: Cellular Diversity and Pathological Feedbacks

The logical framework has also been applied to the simulation of signaling pathways involved in lymphoid related-diseases, like acute lymphoblastic and T cell large granular lymphocyte (T-LGL) leukemia. In the first case, it was predicted that a proinflammatory microenvironment may induce instability in two molecular axes responsible for the retention of hematopoietic progenitor cells within regulatory bone marrow niches (120). In the second case, the model helped to decipher some of the molecular mechanisms that promote survival in T-LGL leukemia cells (121). Both models integrated microenvironmental factors with signaling pathways participating in cellular fate decisions, and in both cases the role of the pro-inflammatory NFkB pathway emerged as important player in the pathogenesis.

Few mathematical models have managed to simulate the dynamic communication between lymphocytes and microenvironment, considering that the feedback loops between both systems are key to modulate immune responses, although the *in vivo* regulation of both systems is more complex due to influence of neighbor tissues and endocrine signals. The perturbation or inadequate coupling of the regulatory interactions among systems have been suggested to trigger inflammation in multiple chronic diseases. For many years the study of pro-inflammatory conditions was focused on the identification of cytokines as biomarkers or target for adjuvant therapies. With the advances on immunotherapy, the study of immune cells as active participants in chronic diseases development and progression has become of great importance because they represent therapeutic targets with less co-lateral effects than conventional therapies.

Recently, it has been probed that epigenetic landscape approach is useful for the *in silico* analysis of health to pathogenic progression (122), such as the epithelial-mesenchymal transition and the induction to migratory phenotype induced after chronic pro-inflammatory conditions, offering a tool to delve deeper into transition stages important for early diagnosis (123–126). Computational modeling of epithelial-mesenchymal transition induced by pro-inflammatory cues has suggested an intermediate stage with a senescent profile (125). The process of epithelial malignant transformation is promoted, among other factors, by TGFβ secreted by CD8 and Treg cells, and TNFα produced by macrophages and pro-inflammatory T cells (127, 128). Importantly, CD8 T lymphocytes have been purposed as players in the promotion of aggressive features in breast cancer tumorigenesis (129); but using CD8 T cells as therapeutic targets implicates affecting one of the most important defenses toward infections, so research about the regulatory networks underlying T cell polarization in dynamic feedback with epithelial cells open new opportunities for the development of more precise therapies by simulating multiple or all the possible perturbations in an integral network as also suggested for breast cancer therapy (130).

The same approach is applicable for the study of emergent attractors from many other networks associated to chronic diseases, for example, type 2 diabetes described in terms of beta-pancreatic cell (115) and T lymphocyte interacting networks, based on evidence of the participation of different T subpopulations as inductors of local and systemic inflammation (131). A first approach targeting CD4 T cell plasticity in metabolic diseases showed that hyperinsulinemia, a condition associated with metabolic syndrome and early stages of type 2 diabetes, inhibits the generation of T regulatory Foxp3 cells and stabilizes the Th17 attractor (10). Besides type 2 diabetes, the increase of Th17 subpopulation and decrease of T regulatory cells have been linked with the destruction of beta-pancreatic cells in the pathogenesis of type 1 diabetes, an increased risk of breast cancer recurrence in diabetic patients and increased susceptibility to develop colitis (132–134). The modulation of the Th17 subpopulation as a promising treatment of colitis was predicted by computational simulations of a continuous model. With *in silico* perturbations of the GRN underlying CD4 T cell differentiation it was predicted that the increase of PPARγ in Th17 cells derives in its transition toward an iTreg profile characterized by the upregulation of Foxp3. The *in vivo* effect of transplanting PPARγ null Th0 lymphocytes was an increased severity and earlier development of colitis in mice. In contrast, pharmacological activation of PPARγ resulted in the induced shift from Th17 to iTreg phenotype that favored colonic tissue reconstitution (135).

The use of integral models of regulatory networks can be also applied to chronic infections. Existing models of infectious diseases and their interconnection with lymphoid regulatory networks are very limited. Even though, one group has reconstructed a logical network to study the intracellular pathways in CD4 T cells affected by the viral proteins during HIV infection. By considering a model composed by 16 viral and 121 CD4 T cell nodes, they predicted new viral-human molecular interactions and obtained conclusions on the signaling flow affecting cellular fate decisions (136).

All chronic processes mentioned above involve multiple developmental stages where different changes in the microenvironment and the cellular composition take place, depending one on the other through feedback loops. With discrete models we can clearly map the stable stages and the transition between them in the presence or absence of particular nodes, while in conventional continuous models it is quite complicated to include as much as transcription regulators are required to simulate cellular transitions of more than one type of cell. So, the transformation of genetic Boolean models using fuzzy logic, is a promising approach to integrate differentiation networks of lymphoid cells and cells from other tissues to construct more accurate models for the study of chronic diseases, as it becomes important the consideration of temporal evolution and graded changes in molecular compositions. Additionally, less frequent inflammatory cells participating in chronic diseases can be included, like in chronic allergic lung disease, where the progressive accumulation of B cells in the lung promotes Th2 responses by the antigen presentation process (137).

Moreover, intermittent or persistent rapid perturbations in chronic and complex diseases, but not during steady states, provoke small and sometimes, cumulative variations within the cells or their environment, including modifications in cytokines secretion patterns, cellular populations proportions, miRNAs expression, etc., that mostly become visible until there is an abrupt transition of the whole system. Of note, fuzzy logic continuous models permit an easy simulation of such periodical and transient signals that are transduced by cell signaling pathways.

**Figure 4:**
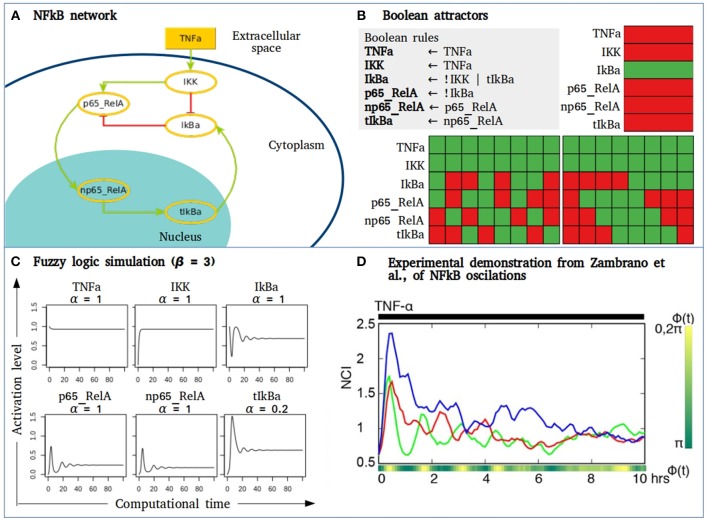
Fuzzy models to study signaling pathways activation. **(A)** NFkB network where IKK is stimulated by microenvironmental TNFα. IKK phosphorylates IkBa unrepressing the dimer p65_RelA to allow its translocation to the nucleus. In the nucleus p65_RelA promotes the transcription of IkBA closing a negative feedback loop of the NFkB pathway. **(B)** The Boolean simulation of the network generates three attractors, two of them are cyclic attractors with TNFα activation. Here, green = 1, red = 0. **(C)** Activation value of the node in the NFkB network obtained by fuzzy logic simulation. In this case, β = 3, and α varies depending on the type of biochemical event in which each node is involved. Node tIkBa represents a transcriptional event, with decay rate α = 0.2. **(D)** Figure taken from (138) showing the nuclear to the cytoplasmic GFP intensity (NCI) of three single GFP-p65 cells stimulated with a constant flow of 10 ng/ml of TNFα.

The utility of fuzzy models may apply to a small network composed by some of the main components of the NFkB signaling pathway that behave as a damped oscillator during activation with TNFa (Figure 4). The Boolean simulation of the NFkB network generates two different cyclic attractors when TNFa is activated. However, when simulating the network as a fuzzy logic and varying the parameter of the “slow” reaction corresponding to the genetic transcription of IkBa, damped oscillations as observed in Zambrano et al., are recorded (138). Without introducing any specific kinetic information of receptor affinity, phosphorylation kinetics or translocation velocity, the fuzzy model shows the transition from an initially perturbed system toward a stable state with a controlled or regulated NFkB activation. As suggested by Zambrano et al., these approaches may aid to understand normal cell responses but also their behavior in diseases like cancer, where NFkB activity is usually disregulated and out of control, driving to multiple biological consequences including hyperproliferation, cell survival or migration.

## 5. Conclusions

Lymphocytes are active participants of many biological processes involved in homeostasis and can evolve concomitantly to tissues transiting through a pathogenic transformation, due to their responsiveness to a large diversity of biochemical signals and their plasticity. *In silico* experimentation with regulatory networks has shown the potential to identify the underlying mechanisms of feedback loops that participate in the promotion of disease progression or in the establishment of chronic inflammation. Additionally, the adaptation of existing models for the study of lymphocytes diversity in pathogenic contexts using powerful tools like fuzzy logics represents an approach to visualize the global effect of potential immunotherapeutics.

## Author Contributions

JE: analysis of published data, discussion of the topic-related information, drafting, and writing the paper. CV and RP: analysis of published data, discussion of the related information, drafting, writing the paper, critical review of the intellectual content.

### Conflict of Interest Statement

The authors declare that the research was conducted in the absence of any commercial or financial relationships that could be construed as a potential conflict of interest.
